# Supplementary material for: Virtual Screening of Soybean Protein Isolate-Binding Phytochemicals and Interaction Characterization
Source: Foods. 2023 Jan 6;12(2):272. doi: 10.3390/foods12020272 (PMC9857816; doi:10.3390/foods12020272)
Supplement: Supplementary file 1 [file foods-12-00272-s001.zip › foods-2080832-supplementary.pdf]

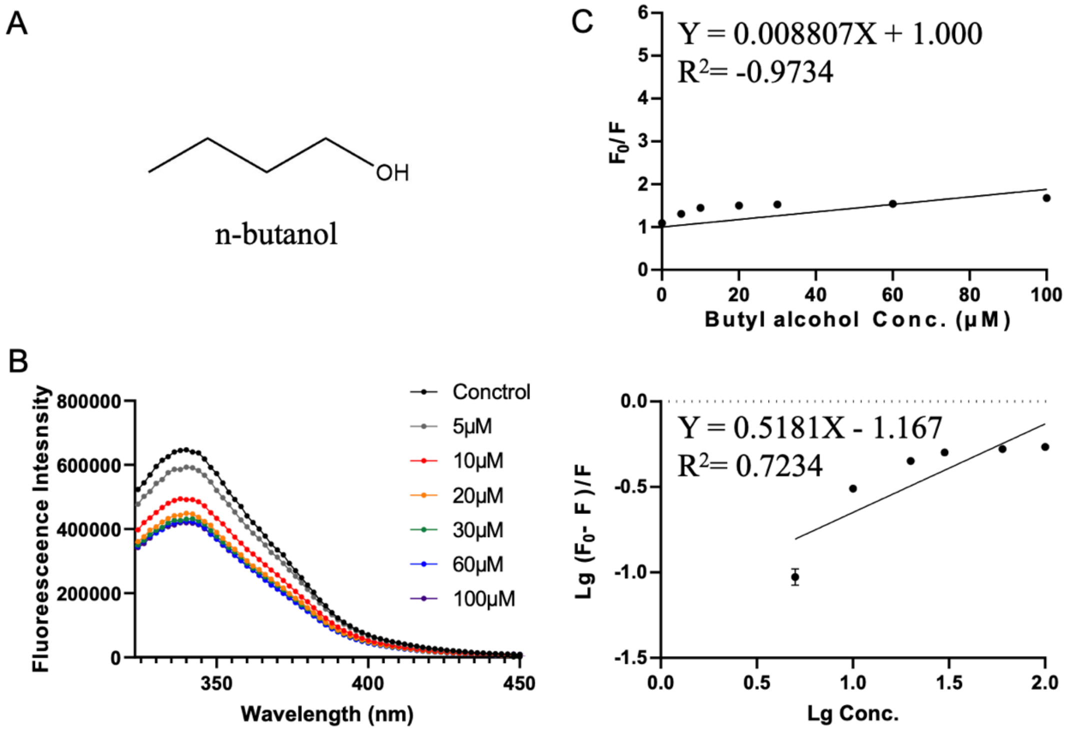

Figure S1. Characterization of SPI-n-butanol interaction by fluorescence quenching assay, as a negative control. (A) Molecular chemical structure of n-butanol. (B) The fluorescence emission spectra of SPI titrated with different concentrations of n-butanol. (C) The Stern-Volmer plots for binding parameter calculation. The obtained F_0/F and $lg[(F_0 - F)/F]$ were not fitted well with equation (2) (Figure S1C, R^2 = -0.9734) and equation (3) (Figure S1C, R^2 = 0.7234) due to the poor concentration dependence.
